# Population genetic structure of the African sugarcane stalk borer (*Eldana saccharina* Walker, Lepidoptera: Pyralidae) in Tanzania

**DOI:** 10.3389/finsc.2026.1806215

**Published:** 2026-05-04

**Authors:** Hamis D. Wambura, Gration M. Rwegasira, Martin J. Martin

**Affiliations:** 1 Department of Agricultural Sciences, Mwalimu Nyerere University of Agriculture and Technology, Butiama, Mara, Tanzania; 2 Department of Crop Science and Horticulture, Sokoine University of Agriculture, Morogoro, Tanzania; 3 Institute of Pest Management, Sokoine University of Agriculture, Morogoro, Tanzania

**Keywords:** *Eldana saccharina*, genetic diversity, haplotypes, population structures, sugarcane pastes

## Abstract

**Introduction:**

The African sugarcane stalk borer (*Eldana saccharina*) is a major insect pest of sugarcane in Sub-Saharan Africa. Because its larvae reside inside sugarcane stalks, conventional measures are less effective. Poorly managed infestations can reduce sugar production by up to 18% from damage caused by a single larva. Limited knowledge on the genetic diversity and population structure of *E. saccharina*, has curtailed insights into developing breeding-based pest management strategies including host resistance.

**Method:**

Samples of *Eldana saccharina* were collected from three altitude-defined agroecological zones in Tanzania. A mitochondrial cytochrome c oxidase subunit I (COI) region was amplified, sequenced, and analyzed to assess genetic diversity, population structure, and phylogenetic relationships among populations.

**Results:**

Twelve unique haplotypes were identified. Observed haplotype diversity was high in the high altitude (Hd=0.64) and medium (Hd=1.00) populations, and moderate in the low-altitude populations (Hd=0.57), although these estimates should be interpreted cautiously due to the small sample size used. Overall genetic differentiation across populations was significant (χ² = 38, P = 0.017). Pairwise comparison showed significant differentiation between high and low altitude populations (FST = 0.35, P = 0.004) and between medium and low altitude populations (FST = 0.21, P = 0.01), whereas differentiation between high and medium altitude populations was low and not significant (FST = 0.1, P = 0.12) A significant positive Tajima’s D value in the low-altitude (2.5, P = 0.01), may indicate possible population contraction or selection, although this inference is provisional due to limited sample size. The presence of geographically structured and zone-specific haplotypes was observed, suggesting preliminary altitude-associated genetic differentiations.

**Discussion:**

*E. saccharina* populations in Tanzania showed substantial mitochondrial genetic diversity and altitude-associated population structure. These findings suggest preliminary basis for considering population variation in the design of locally relevant monitoring, host-resistance breeding, and other targeted pest management strategies.

## 1 Introduction

Sugarcane (*Saccharum officinarum* L.) is the second highly produced crop worldwide after cereals (1). The crop contributes 80% of the required sugar in the world (2), with Brazil being the leading sugarcane-producing country worldwide, contributing 39% of the produced sugarcane (1). In Africa, South Africa is the largest sugarcane producer, contributing 18.4 million metric tons per annum, while Tanzania is ranked the 9th on the list, contributing 3.4 million metric tons of sugarcane (3). Despite the economic importance of sugarcane, the crop is highly affected by several insect pests, the African sugarcane stalk borer (*Eldana saccharina* Walker) being the most destructive (4). The pest also causes a 5%–18% sugar reduction by boring into the sugarcane stalk, hindering the normal flow of water and nutrients, and in turns results in to significant sucrose losses (5). Sugarcane fields in Tanganyika Planting Company Limited (TPC) in Kilimanjaro Region, Tanzania, were the first reported to be infested with *E. saccharina* (6). Recently, TARI (7) declared the pest infests the crop in fields owned by other large sugarcane-producing companies in the country, namely, Kagera Sugar Limited, Kilombero Sugar Limited, and Mtibwa Sugar Estates, located in the Kagera and Morogoro Regions.

Increasing incidences of *E. saccharina* continues to threaten the productivity of sugarcane industries (7, 8). Moreover, effective management of the pest might not be possible without understanding the genetic diversity of existing species. Integrating aspects of breeding for resistance to the pest as a vital component of Integrated Pest Management strategies remains imperative. Thus, the need for knowledge on the diversity of *E. saccharina* as a basis of a management strategy may not be overemphasized. Peng et al. (9) observed that genetic variation within and between populations can influence insects’ key biological traits such as host preference, environmental adaptation, and resistance to management interventions. Other studies have reported substantial genetic differentiations among *E. saccharina* populations, largely attributed to distinct ecological conditions across regions such as South Africa, Benin, Cameroon, Uganda, and Ethiopia (8, 10). Similarly, (8) demonstrated that environmental heterogeneity contributes to genetic variability in *E. saccharina* individuals, affecting its distribution and abundance in South Africa, Benin, Uganda, Ethiopia, Ghana, Mozambique, Zimbabwe, Nigeria, and Senegal. Despite these findings, the extent to which genetic variation shapes the diversity, structure, and differentiation of *E. saccharina* populations in Tanzania remains poorly understood. Despite the presence of the recent reports that confirm the presence of the pest in major sugarcane-producing fields across different agroecological zones in the country (7), several knowledge gaps still exist, particularly on the biological and ecological parameters of the pest, including its genetic composition.

The diverse agroecological zones that exist in Tanzania (11) vary in temperature, relative humidity, rainfall amount and distribution, as well as vegetation covers (12). Elsewhere, these attributes have shown direct influence on the genetic composition of different insect populations (13–15). The variations of the environmental factors and host plant distribution are mentioned to drive gene flow among *E. saccharina* populations (8). Additionally, effective crop pest management is well known to depend on understanding genetic structure, as genetic differentiation influences dispersal and responses to control strategies (16). *E. saccharina* is among the most destructive sugarcane insect pests in Sub-Saharan Africa, causing major sugarcane losses in the region (2, 17–19). These impacts highlight the need to assess the pest’s genetic structure to support improved management approaches. Thus, determining the genetic diversity across these agroecological zones in Tanzania remains important to the understanding of population connectivity and evolutionary relationships. This study, therefore, aimed at assessing the genetic diversity of *E. saccharina* was collected from three distinct (low, medium, and high altitude) agroecological zones in Tanzania. The findings provide initial insights into the mitochondrial genetic structure of *E. saccharina* in Tanzania may help inform the development of more sustainable and locally relevant management strategies against this sugarcane pest.

## 2 Materials and methods

### 2.1 Description of the study area

**Figure 1:**
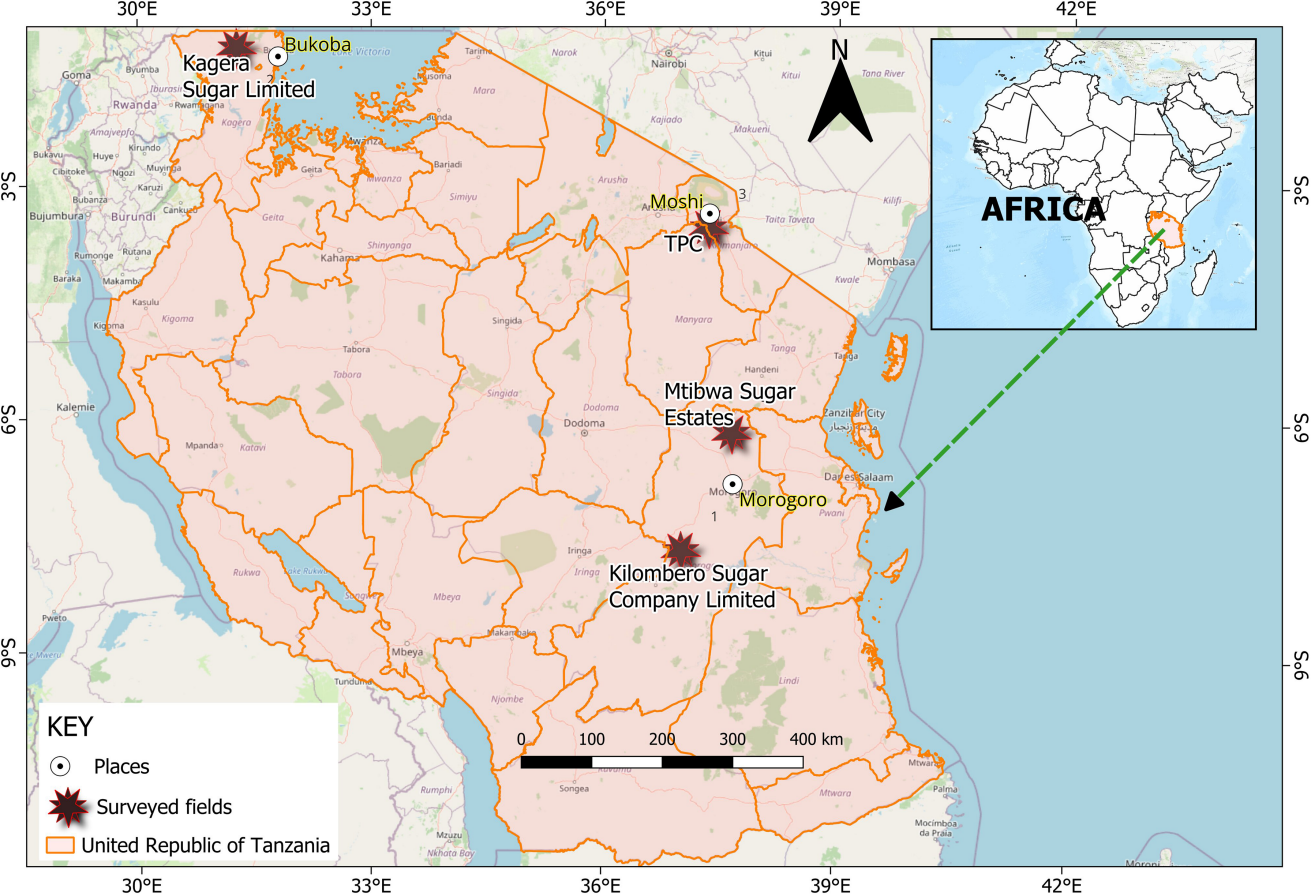
A map of the study area showing the geographic locations of *E. saccharina* sampling areas across the three agroecological zones in Tanzania: high altitude (Kagera sugar limited), medium altitude (TPC), low altitude (Kilombero sugar company limited and Mtibwa sugar estates).

Adults of *E. saccharina* were collected from fields owned by the four major sugarcane-producing companies in Tanzania (**Figure 1**), representing distinct altitudinal zones classified as low (<600 m a.s.l), medium (601–1000 m a.s.l), and high (>1,000 m. a.s.l) altitude areas (20). The high-altitude area was represented by Kagera Sugar Limited, located in Bukoba District, Kagera Region, in North-Western Tanzania, at approximately 1400 m a.s.l. This region experiences temperatures ranging from 20 °C to 28 °C and receives biannual rainfall varying between 500 and 2000 mm annually. The medium-altitude zone was represented by TPC in Moshi District, Kilimanjaro Region, North-eastern Tanzania, situated at an average altitude of 950 m a.s.l. Annual temperatures range from 17 °C to 29 °C, with a bimodal rainfall pattern (600–2100 mm) whereby long rains are experienced from March to May and short rains from October to December annually (21). Low-altitude areas were represented by Kilombero Sugar Company and Mtibwa Sugar Estates Limited, both located in Morogoro Region, Eastern Tanzania, at an average altitude of 539 m a.s.l. This region experiences temperatures of 18 °C–30 °C, bimodal rainfall ranging from 600–1800 mm annually, and a dry season occurring between July and September (22).

### 2.2 Sampling protocols

A total of 24 adult *E. saccharina* were collected from fields owned by the four sugarcane-producing companies in Tanzania, including Kagera Sugar Limited (high altitude), Tanganyika Plantation Company (medium altitude), Kilombero Sugar Company, and Mtibwa Sugar Estates (low altitude). The sampling was conducted on multiple dates between 15 February and 30 April 2025 across the study areas, with site-specific collections undertaken at Kagera Sugar Limited from 15 February to 3 March 2025, at Tanganyika Plantation Company from 24 March to 7 April 2025, and at Kilombero Sugar and Mtibwa Sugar Estates from 17 to 30 April 2025. Although the target sample size was 60 adults, with 20 samples per agroecological zone, low infestation levels in the low-altitude areas led to only eight adults (six from Kilombero and two from Mtibwa) being collected from the low-altitude fields. Thus, a total of eight individuals per altitude category were retained for this study, corresponding to the smallest available sample size. This standardization was used to facilitate direct comparison among altitude categories and reduce bias associated with unequal sample sizes in genetic diversity and population structure analyses, resulting in a total sample size of 24 (23). This sample size was also retained for an exploratory mitochondrial survey, although it limits statistical power and warrants cautious interpretation of genetic diversity and demographic estimates (24).

Sampling sites within sugarcane fields were determined using a cardinal sampling technique, as described by Marchioro & Faccoli (25)Minarsch et al. (26), and Montgomery et al. (27). Block disposition maps were obtained from each company, and two blocks per cardinal direction were randomly selected. Within each block, diagonal transect lines were established, along which light traps with 2 m × 3 m sheets equipped with UV-A blacklight or white/LED fluorescent bulbs were deployed at 100 m intervals during the night between 19:30h and 20:30h), following the method by Truxa & Fiedler. (2012). On average one trap per hectare was considered.

In the high-altitude cluster (Kagera Sugar Limited), 122 sampling sites were established across four cardinal points. The north had two 20-ha blocks with 20 light traps per block, the south had 16 and 20 traps in 16-ha and 20-ha blocks, the west had two 15-ha blocks with 15 traps each, and the east had 16 and 20 traps in 16-ha and 20-ha blocks. The medium altitude (TPC) followed a similar approach, totaling 124 sampling sites distributed among four cardinal points with varying block sizes (10–21 traps per block).

In the low-altitude cluster, represented by Kilombero Sugar Company and Mtibwa Sugar Estates, a total of eight blocks were sampled. Kilombero had 104 traps across northern, southern, western, and eastern blocks ranging from 22 to 34 traps, while Mtibwa had 12, 8, 13, and 16 traps in blocks of 8–16 ha. Although 399 trapping sites were established across the three agroecological zones to maximize adult recovery, these sites represent sampling effort rather than the number of genetic samples analyzed. A summary of replication and experimental design is presented in **Table 1**.

**Table 1:** Summary of replications and experimental design.

Scale of inference	Scale at which the factor of interest is applied	Number of replicates at the appropriate scale
Individual	3 agroecological zones	High, medium and low altitudes
Individual	Sampled blocks	4 blocks/altitude
Individual	Number of light traps	One trap at every 1 ha in a block
Individual	Collection days	5 days/trap/block
Individuals	Agroecological zones	8 samples per altitude

To increase trapping efficiency, both UV-A blacklight and white light traps (20 W each) were alternated (28). Adults captured in traps were collected with aspirators and immediately preserved in 98% ethanol for effective dehydration of sample tissues and reduced DNA hydrolysis (29). Samples were transported in cool boxes with ice packs to the Entomology Laboratory at Sokoine University of Agriculture, where they were stored at −20 °C prior to morphological examination and molecular analyses. The fact that the systematic sampling technique deployed ensured coverage of all agroecological zones; it was a reliable and true representation of *E. saccharina* population in Tanzania, upon which the preliminary information on the structure and genetic diversity of the insect could be established. Although the sampling design provided broad coverage across the three agroecological zones, the limited number of adults collected, particularly in the low-altitude mean that the resulting genetic dataset should be interpreted as preliminary rather than fully representative of *E. saccharina* populations in Tanzania (24).

### 2.3 Morphological identification of the collected samples of *E. saccharina*

Identification of the collected samples to the species level as *Eldana saccharina* was conducted using both online and physical published morphological identification keys (5, 30). The distinctive characteristics that differentiated *E. saccharina* from other sugarcane borers was used for characterization and identification. These included forewings with pale brown color, two dark spots in the anterior half of each forewing, brown longitudinal veins and a short fringe in the hindwings, and the presence of roof-like forewings. After the clear verification that the insect pest under study was *E. saccharina* morphologically, the samples were then taken to the biotechnology laboratory at Sokoine University of Agriculture for further molecular identification.

### 2.4 DNA extraction and amplification

The total DNA from each sample was extracted from the thoracic tissue following the protocol in the Qiagen DNeasy Blood and Tissue kit. Polymerase chain reaction (PCR) amplification targeted a fragment of the mitochondrial cytochrome c oxidase subunit I (COI) gene using the forward primer Ron-V (5′-GGAGCTCCAGATATAGCTTTCCCC-3′) and reverse primer K525 (-5’-ACTGTAAATATATGATGAGCTCA-3′) following Assefa et al. (4). The primers were synthesized by Humanizing Genomics Macrogen (ligo™), and all downstream population-genetic analyses were based on this mitochondrial COI locus. Amplification was carried out in a reaction volume of 50 µl consisting of 31.25 µl of 2× master mix, 2.5 µl forward primer, 2.5 µl reverse primer, 12.55 µl of nuclease-free water, and 1.2 µl of the sample DNA. The PCR machine (GeneAMP PCR System 9700) was used to perform 35 cycles under the denaturation condition of 94 °C for 30 s, an annealing temperature of 55 °C for 45 s, an elongation temperature of 68 °C for 1 min, and a final elongation temperature of 68 °C for 6 min.

The visualization of amplified DNA bands was by gel electrophoresis whereby 1 g of agarose powder was weighed using a sensitive weighing balance (Sartorius AG Germany CPA 124S) and then added to 99 ml of 1× TAE (Tris-Acetate EDTA) buffer. The mixture was heated in a microwave for 2 min to completely dissolve the agarose powder, after which the gel was taken out of the microwave, cooled, and 1 µl of stain (ethidium bromide) was added to give colors to DNA bands. The gel was then poured into a casting tray and left for 20 min for gel solidification. To provide a molecular size reference, the 100 bp DNA ladder from New England Biolabs was loaded alongside the samples from the PCR machine (5 µl each) in a prepared gel, and then the electrophoresis was allowed to run for 1:30h. The DNA bands were then visualized in a UV transilluminator machine, in which samples that showed a clear band migrating at approximately 650 bp were considered to have yielded successful amplification of the targeted mitochondrial DNA fragment.

### 2.5 Sequencing and sequence analysis

The PCR products corresponding with positively amplified samples were sent to Macrogen Europe in the Netherlands for DNA sequencing. The AB1-formatted DNA sequences for *E. saccharina* received from the Macrogen laboratory were singly blasted onto National Center for Biotechnology Information (NCBI) for species comparison. Only the DNA sequences that matched other *E. saccharina* sequences in the database by 94%–100% were considered for analysis. Although numerous adult moths were collected from high and medium altitudes, only eight adults were obtained from low altitudes. In addition, some field-collected specimens that were morphologically similar to *E saccharina* were excluded after sequencing because their DNA sequences did not confirm species identity. Consequently, the final DNA sequence dataset was restricted to sequences confirmed as *E. saccharina* individuals. Because the low altitude yielded only eight confirmed individuals, the analyzed dataset was standardized to eight individuals per altitude to facilitate direct comparison across zones to reduce bias associated with unequal sample size (23). Finally, from high and medium altitudes, the eight retained DNA sequence products were selected from sequence-confirmed specimens with successful amplification and high-quality reads while maintaining coverage across sampling sites. Sequencing generated fragments with an average raw length of 472 bp, in which prior to analysis, sequences were edited and trimmed to remove low-quality bases and non-overlapping regions. The quality of selected DNA sequences was checked using sequence scanner software (Thermo Fisher Scientific, 2025), in which DNA sequences with sharp and well-separated peaks were selected. The sequences with the quality value (QV) ranging from 30% to 99.9% were considered for analysis while <30 QV sequences were trimmed out (31). The cleaned 24 DNA sequences were then aligned by CrustalW method in MEGA12 software. The misaligned regions and excessive gaps were manually deleted. The final aligned DNA sequences (357 bp) were then saved into FASTA file format for further phylogenetic analysis. Because the analysis was based on a single mitochondrial locus, the resulting genetic inferences were treated as preliminary and interpreted cautiously.

To determine the DNA polymorphism for *E. saccharina* populations in Tanzania, the 24 aligned sequences were subjected to DNA Sequence Polymorphism (DnaSP) software version 6.12.03 for estimating sequence variation and identifying haplotypes. A median-joining haplotype network was then constructed and visualized in PopART computer software to illustrate genealogical relationships among the identified haplotypes. To infer the evolutionary relationship of *E. saccharina* populations and allowing easy assessment of phylogenetic clustering by agroecological zones, the maximum likelihood (ML) phylogenetic tree was generated in the IQ-TREE computer program. Prior to tree reconstruction, the best-fit nucleotide substitution model was selected automatically in the IQ-TREE using the Bayesian Information Criterion (BIC), with HKY+F+I identified as the optimal model for the aligned DNA dataset. Branch support was assessed using 1,000 ultrafast bootstrap replicates. HKY+F+I was identified by the program as the best fit substitution model for phylotype clustering. The resulting ML tree was subsequently visualized in FigTree computer application (version 1.4.4).

The analysis of molecular variance (AMOVA) was performed in Arlequin to determine the genetic differentiations within and between *E. saccharina* populations. In which the aligned 24 DNA sequences in FASTA format were categorized into high, medium, and low-altitude clusters. The hierarchical population structure was then converted to the Arlequin format (.arp file extension) and subjected into the Arlequin computer running software. The AMOVA was then run in Arlequin to which genetic variations within and between *E. saccharina* populations were displayed. The statistical significance of the observed genetic variations was observed by performing a pairwise F_ST_ test at *p* ≤ 0.05. Genetic distances for the DNA sequences within and between *E. saccharina* populations were calculated in the R statistical package, and the resulting distance matrix was analyzed using principal coordinates analysis (PCoA) to assess and visualize genetic clustering among individuals from the sampled agroecological zones. Permutation multivariate analysis of variance (PERMANOVA) was further performed in R statistical software to confirm the statistical significance of the observed genetic variations. The R scripts for running PERMANOVA for the DNA sequences in FASTA file format were prepared. The metadata consisting of sample names and zones was also prepared. The two files were then subjected to R software using “ape and vegan packages” for PERMANOVA. The Kimura-2-parameter (K2P) was used to calculate the pairwise genetic distance matrix for the *E. saccharina* DNA sequences in 9,999 permutations. The *post-hoc* of the PERMANOVA results was then conducted to test the significance of the pairwise comparison between high and medium altitudes; high and low altitudes; and medium and low altitudes. The adjusted *p*-values were obtained using the false discovery rate (FDR).

To determine whether *E. saccharina* populations in Tanzania is stable or has undergone expansion or contraction; the population demographic analysis was carried out in DnaSP software. In addition, a neutrality test was also performed in DnaSP software to assess whether the observed genetic variations of *E. saccharina* fits the expectations of the neutral theory that assumes most mutations are selectively neutral. Therefore, the aligned DNA sequences were defined based on their agroecological zones and then subjected to DnaSP computer software to conduct a mismatch distribution that compared the observed distribution of pairwise differences among sequences to expected distributions under expansions, contractions, or stability. To achieve this, the Tajima’s D and Fu’s Fs values were calculated to establish their statistical significance at *p* ≤ 0.05. The Mantel test was performed using the genetic distance matrix in DnaSP computer software to evaluate whether the observed genetic differentiations among *E. saccharina* populations are associated with geographic or environmental distances. Additionally, the Mantel test was performed to determine whether the genetic structure of *E. saccharina* individuals are influenced by spatial separation and altitude differences. Statistical significance in gene flow and isolation by distance among *E. saccharina* populations were observed at *p* ≤ 0.05 using 9,999 permutations.

Patterns of genetic similarity and divergence of genetic distances of *E. saccharina* across the three agroecological zones was visualized using a PCoA performed in R statistical software. The PCoA also enabled the visualization of genetic structuring of *E. saccharina* across zones. Therefore, in R statistical software (version 4.5.1), the statistical packages with their respective libraries for PCoA, including apes, adegenet, factoextra, ggplot2, ade4, and dplyr, were installed. The aligned DNA sequences were defined in their respective altitudes and loaded in R. The genetic distances were then generated using the Kimura-2-Parameter (K2P) model of nucleotide substitution. Thus, the generated genetic distances were loaded and read in R, where the PCoA graph was plotted for visualization and interpretation.

## 3 Results

### 3.1 Similarities of *E. saccharina* populations in Tanzania

The BLAST analysis of mitochondrial DNA sequences from the three agroecological zones showed a high similarity to the existing sequences in the NCBI GeneBank database (E-value = 0.0). The percentage identity ranged from 94.76% to 100% (**Table 2**).

**Table 2:** BLAST search of DNA sequences similar to the Tanzanian *E. saccharina* isolates.

Sample ID (present study)	Closest match organism	Identical accession number	Country (origin)	Quary coverage (%)	Identity (%)	E-value
KGR-1	*Eldana saccharina*	DQ486925.1	Uganda	99	100	0.0
KGR-2	*Eldana saccharina*	DQ486910.1	Uganda	98	100	0.0
KGR-3	*Eldana saccharina*	DQ486923.1	Senegal	98	100	0.0
KGR-4	*Eldana saccharina*	DQ486923.1	Senegal	98	100	0.0
KGR-5	*Eldana saccharina*	DQ486923.1	Senegal	99	100	0.0
KGR-6	*Eldana saccharina*	DQ486925.1	Uganda	99	100	0.0
KGR-7	*Eldana saccharina*	DQ486922.1	Senegal	99	100	0.0
KGR-8	*Eldana saccharina*	DQ486924.1	Uganda	99	100	0.0
TPC-1	*Eldana saccharina*	DQ486909.1	South Africa	98	94.76	0.0
TPC-2	*Eldana saccharina*	DQ486909.1	South Africa	98	97.06	0.0
TPC-3	*Eldana saccharina*	DQ486926.1	Benin	97	97.32	0.0
TPC-4	*Eldana saccharina*	DQ486909.1	South Africa	97	97.49	0.0
TPC-5	*Eldana saccharina*	DQ486926.1	Benin	97	97.29	0.0
TPC-6	*Eldana saccharina*	DQ486925.1	Uganda	98	100	0.0
TPC-7	*Eldana saccharina*	DQ486909.1	South Africa	99	97.21	0.0
TPC-8	*Eldana saccharina*	DQ486909.1	South Africa	96	97.77	0.0
MTB-1	*Eldana saccharina*	DQ486910.1	Uganda	99	99.76	0.0
MTB-2	*Eldana saccharina*	DQ486909.1	South Africa	99	97.76	0.0
MTB-3	*Eldana saccharina*	DQ486910.1	Uganda	99	99.76	0.0
MTB-4	*Eldana saccharina*	DQ486923.1	Senegal	99	97.76	0.0
KLM-1	*Eldana saccharina*	DQ486910.1	Uganda	99	99.76	0.0
KLM-2	*Eldana saccharina*	DQ486923.1	Senegal	99	97.76	0.0
KLM-3	*Eldana saccharina*	DQ486910.1	Uganda	99	99.76	0.0
KLM-4	*Eldana saccharina*	DQ486909.1	South Africa	99	97.76	0.0

### 3.2 Genetic variation of *Eldana saccharina* populations

A total of 24 sequences were obtained from *E. saccharina* samples collected from Kagera, Kilimanjaro, and Morogoro Regions of Tanzania, with an average raw sequence length of 474 bp. After sequence editing, trimming, and alignment to a common overlapping region, a final aligned length of 357 bp was retained for analysis. Twelve haplotypes were identified from the 24 DNA sequences across the three populations (**Table 3**), in which four haplotypes were from samples collected from Kagera Sugar Plantations in Kagera Region; eight haplotypes were for samples collected from TPC in Kilimanjaro Region; while two haplotypes were from samples collected at Kilombero and Mtibwa sugar estates, both in Morogoro Region. Most haplotypes appeared once in a population, except for haplotype numbers 1, 4, and 12, which appeared four times each in a population (**Table 3**). After alignment, the *E. saccharina* fragments had sequence lengths of 357 bp with 34 parsimony informative sites. The base composition for the gene was 31% Adenine (A), 14.9% Cytosine (C), 13.1% Guanine (G), and 41% Thiamine (T). Adenine and Thymine (AT) composition was recorded to be 72%, while Guanine and Cytosine (GC) composition was 28%. Net nucleotide divergence (Da) among populations was higher between the high and medium altitude clusters (Da 0.02), while DNA sequences from the high-altitude cluster diverged from DNA sequences from the low-altitude population cluster by Da 0.01. Additionally, a very low sequence divergence of Da 0.007 was recorded between medium and low-altitude clusters. The overall haplotype diversity (Hd) and nucleotide diversity (π) for the 24 samples were 0.89 and 0.03, respectively. The haplotype and nucleotide diversities per population clusters are presented (**Table 4**).

**Table 3:** Identified *Eldana saccharina* haplotypes in Tanzania.

Haplotype number	High-altitude population cluster frequency (1163 m a.s.l)	Medium-altitude population cluster frequency (746 m a.s.l)	Low-altitude population cluster frequency (331 m a.s.l)
1	4	1	0
2	1	0	0
3	1	0	0
4	1	0	4
5	0	1	0
6	0	1	0
7	0	1	0
8	0	1	0
9	0	1	0
10	0	1	0
11	0	1	0
12	0	0	4

**Table 4:** Genetic diversity for the identified *E. saccharina* populations haplotypes in Tanzania.

Population	Haplotype diversity (Hd)	Nucleotide diversity (π)	Theta (θ)
High altitude	0.64	0.009	0.009
Medium altitude	1.00	0.026	0.032
Low altitude	0.57	0.027	0.018

### 3.3 Distribution of *E. saccharina* haplotypes

**Figure 2:**
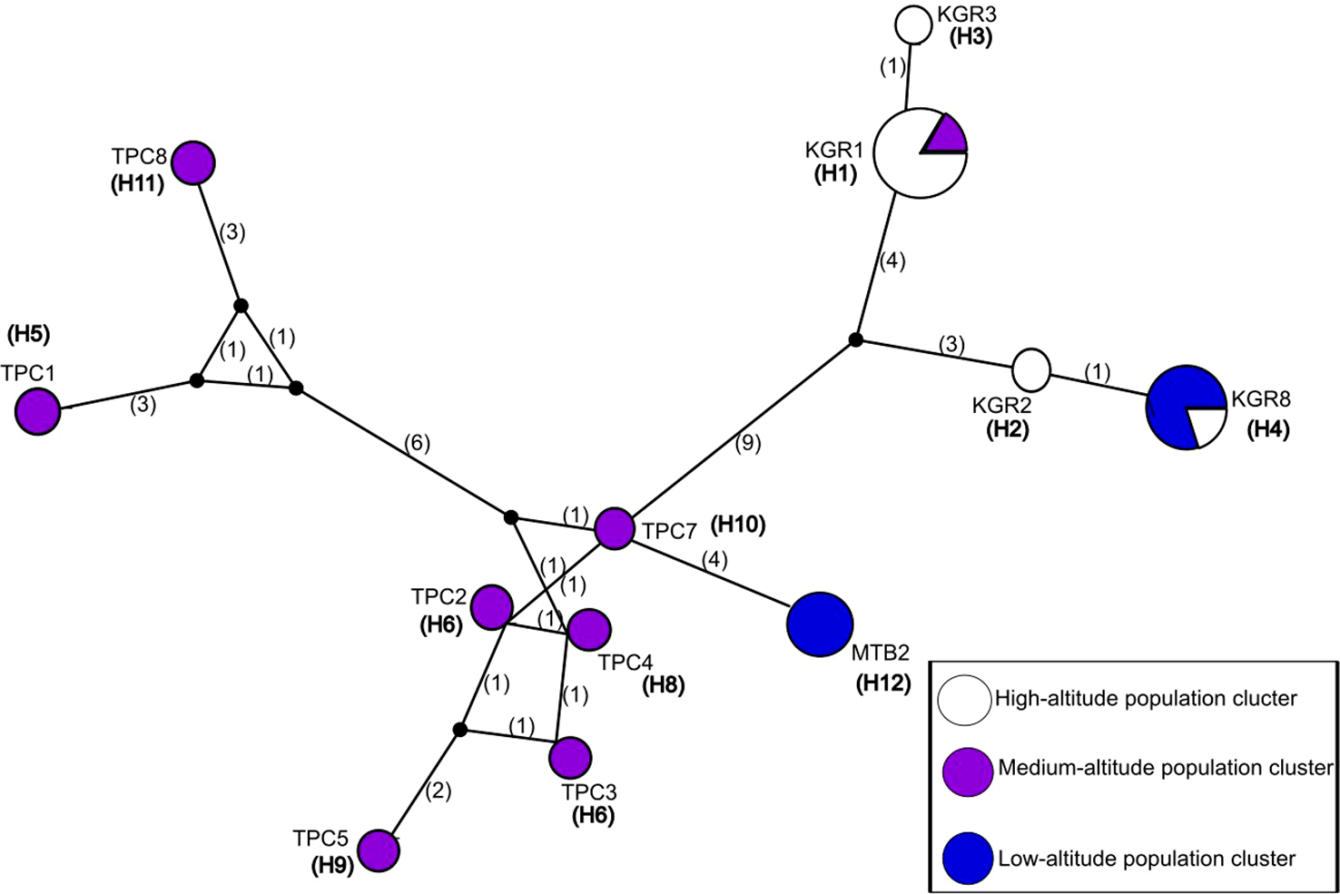
Median-joining haplotype network of *E. saccharina* populations in Tanzania. Each circle represents a haplotype, and circle size is proportional to the number of individuals sharing that haplotype. Different colors indicate agroecological zones, whereas multicolored circles denote haplotypes shared among zones. Connections between circles represent mutational steps among haplotypes.

The identified 12 haplotypes created a reticulate Median Joining Network with one to six mutational steps (**Figure 2**). Most haplotypes were unique to single population clusters, whereas H1, H4, and H12 were shared among populations (**Table 5**). Two haplotypes in the high-altitude population and one haplotype in the low-altitude population were represented by multiple individuals, while nearly all medium-altitude individuals had unique haplotypes; only TPC6 shared a haplotype with the high-altitude population (**Table 5**).

**Table 5:** Shared and unique haplotypes of *E. saccharina* population.

Haplotype	Sample names	Population cluster
1	KGR1, KGR4, KGR5, KGR6, KGR7, TPC6	High-altitude
2	KGR2	High-altitude
3	KGR3	High-altitude
4	KGR8, MTB1, MTB3, KLM1, KLM3	High/Low altitude
5	TPC1	High-altitude
6	TPC2	High-altitude
7	TPC3	Medium-altitude
8	TPC4	Medium-altitude
9	TPC5	Medium-altitude
10	TPC7	Medium-altitude
11	TPC8	Medium-altitude
12	MTB2, MTB4, KLM2, KLM4	Low altitude

### 3.4 Evolutionary relation of *E. saccharina* populations in Tanzania

**Figure 3:**
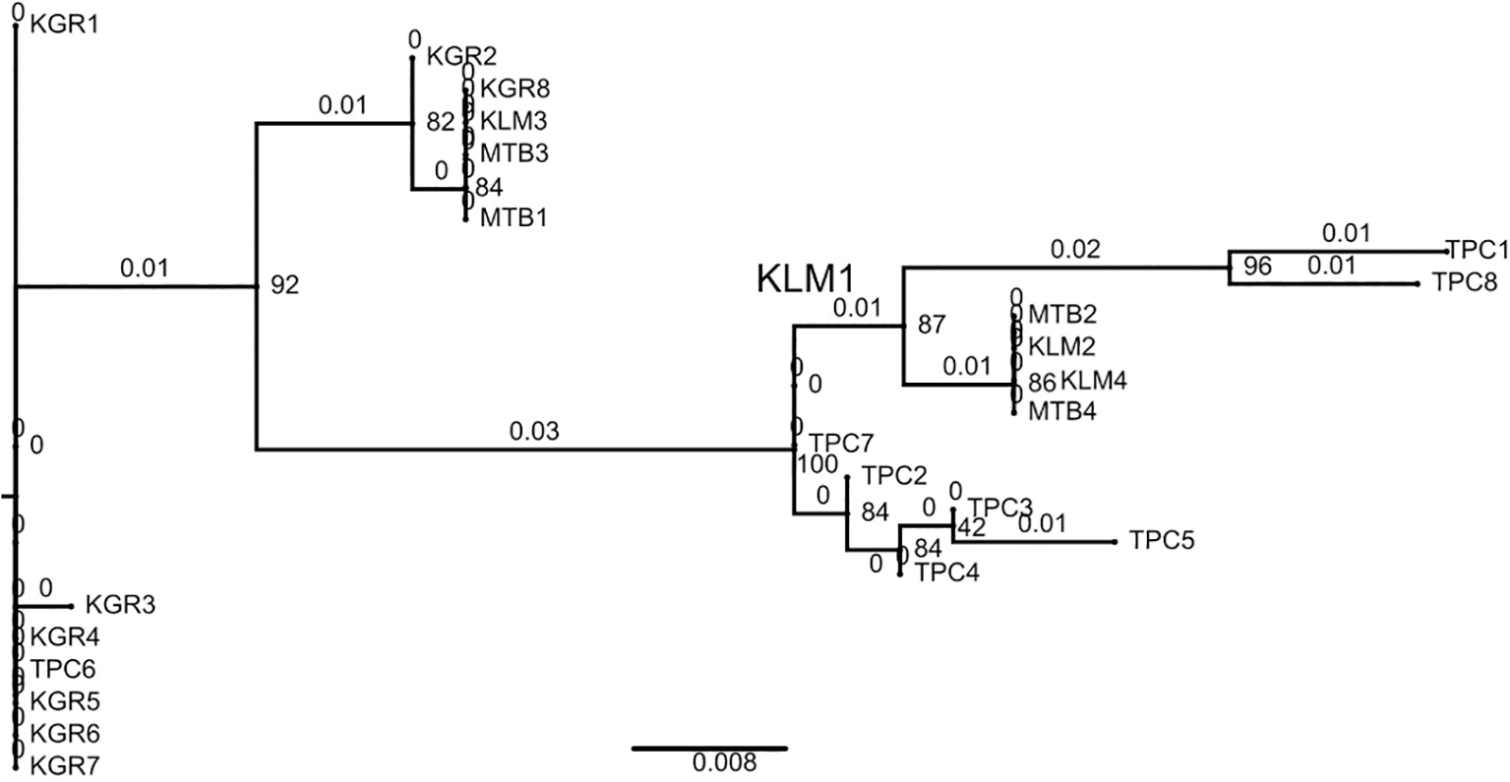
Maximum-likelihood phylogenetic tree of *E. saccharina* sequences from Tanzania generated in IQ-tree to show relative clustering among sequences across agroecological zones. Node values represent ultrafast bootstrap support.

The evolutionary relationship among *E. saccharina* mitochondrial DNA sequences were inferred using a ML approach in IQ-Tree. The existence of ten clades (**Table 6**), of which six were strongly supported (bootstrap values >70%) and four were weakly supported (bootstrap values <70%). One sample from the medium-altitude population (TPC6) grouped within a clade containing four samples from the high-altitude population (**Figure 3**). Overall, sequence grouping largely corresponded to population origin, except in two clades. The major clades were composed mainly of sequences from medium- and low-altitude populations, whereas most high-altitude sequences occurred in minor clades. The phylogeny indicated low genetic divergence among sequences, with a scale bar of 0.008 substitutions per site, and most branches exhibiting short lengths (<1).

**Table 6:** Maximum likelihood (ML) produced clades for *E. saccharina* populations.

Clades	Sample names	Bootstrap values (%)	Remarks
1	KGR1	0	Minor
2	KGR3, KGR4, TPC6, KGR5, KGR6, and KGR7,	0	Minor
3	KGR2	82	Major
4	KGR8, KLM3, MTB3, KLM1, and MTB1	84	Major
5	TPC7	100	Major
6	TPC2	84	Major
7	TPC4	84	Major
8	TPC3, and TPC5	42	Minor
9	MTB2, KLM2, KLM4, MTB4	86	Major
10	TPC1, and TPC8	96	Major

### 3.5 Population structure and differentiation of *E. saccharina*

**Figure 4:**
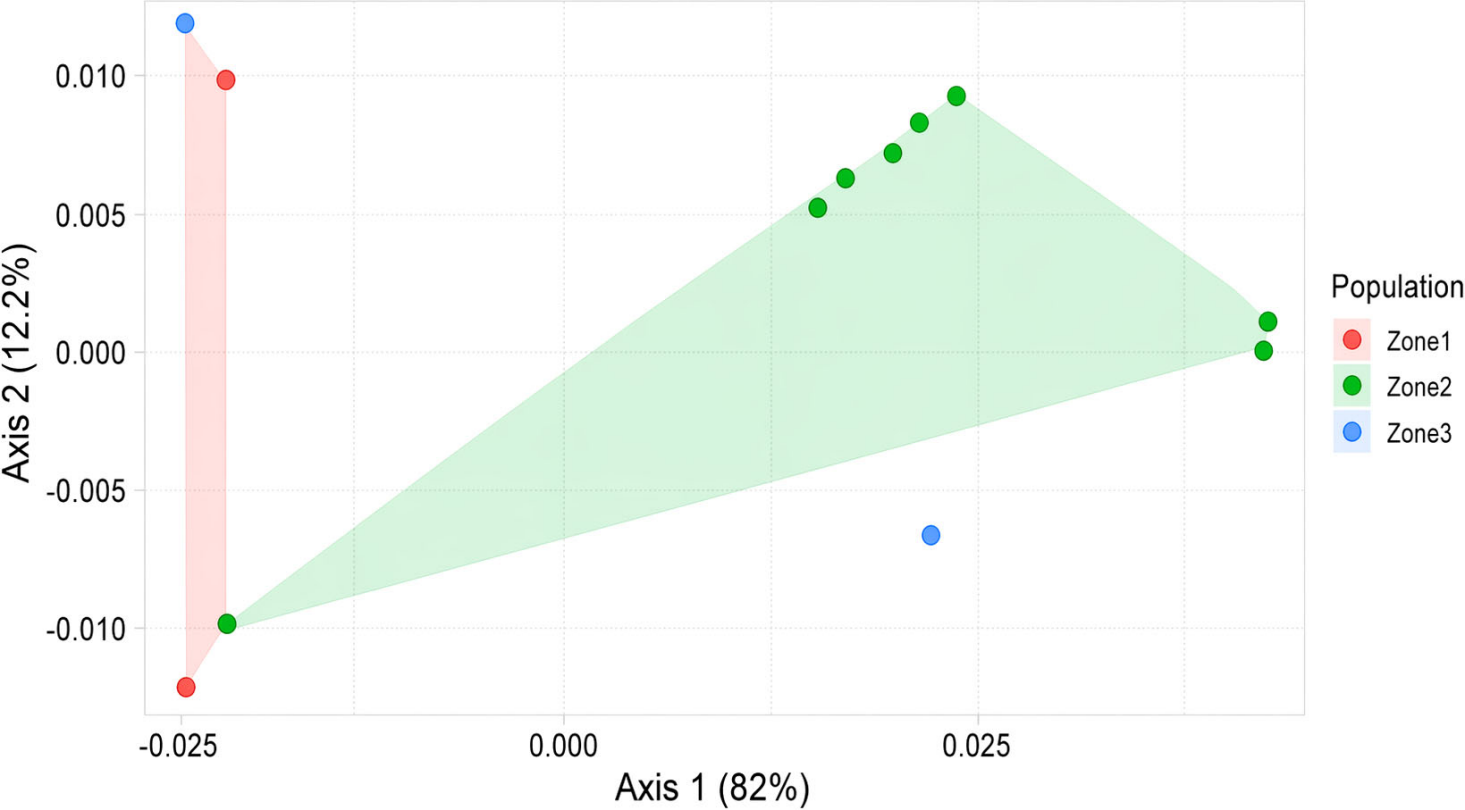
Principal coordinates analysis (PCoA) of mitochondrial DNA variation in *E. saccharina* individuals from Tanzania. Red points represent the high-altitude population (zone 1), green points represent the medium-altitude population (zone 2), and blue points represent the low-altitude population (zone 3). Distances among points reflect genetic variations among individuals.

Genetic analyses revealed significant genetic differentiation among *E. saccharina* populations in Tanzania. A chi-square test indicated overall significant genetic differentiation χ² = 38, *P* = 0.017). Pairwise comparisons showed high genetic differentiation between high and low altitude populations (FST = 0.35, *P* = 0.004) and between medium and low altitude populations (FST = 0.21, *P* = 0.01), while differentiation between high and medium altitude populations was moderate (FST = 0.1, *P* = 0.12). Analysis of molecular variance (AMOVA) indicated strong genetic structuring (Phi (Φ) = 0.23), with 77.44% of the total variation occurring within populations and only 22.56% occurring among populations (**Table 7**). The PCoA of the genetic distance matrix also showed a genetic variation within and between *E. saccharina* populations (**Figure 4**). The first two axes explained 94.2% of the total variation, with Axis 1 accounting for 82% and Axis 2 accounting for 12.2% (**Figure 4**). High- and low-altitude individuals showed partial overlap, whereas medium-altitude individuals were more clearly separated along Axis 1. This pattern was further supported by permutational multivariate analysis of variance (PERMANOVA), which showed significant differences (*P* = 0.001) in genetic variation among the three populations (**Table 8**).

**Table 7:** Analysis of molecular variance in *E. saccharina* samples from three populations.

Source of variation	Degree of freedom	Sum of squares	Mean squares	Variance of component (σ^2^)	% variance
Among populations	2	4.92	2.46	0.22	22.56
Within population	21	15.5	0.74	0.74	77.44
Total	23	20.42	0.89	0.96	100.00
Phi (Φ)					0.23

**Table 8:** PERMANOVA for genetic variations of *E. saccharina* populations.

Source of variation	df	Sum of squares	R^2^	F-value	P-value
Model	2	0.007	0.44	8.35	0.001^***^
Residual	21	0.009	0.56		
Total	23	0.016	1.00		

### 3.6 Demography and neutrality tests for *E. saccharina* populations

**Figure 5:**
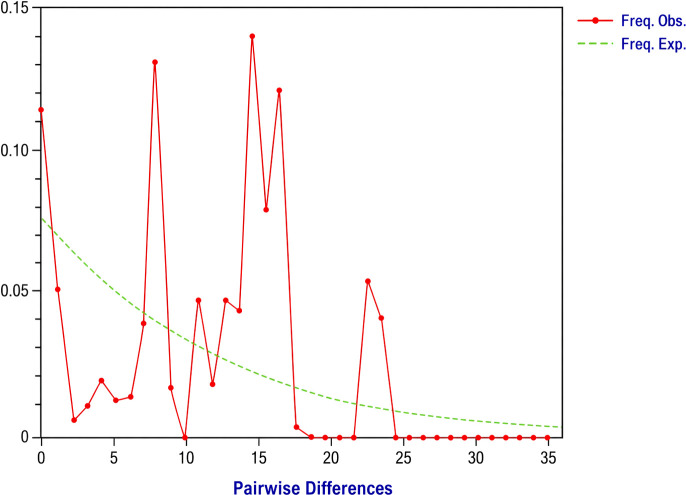
Mismatch distribution of *E. saccharina* populations in Tanzania showing the frequency of pairwise nucleotide differences.

The neutrality test revealed a contrasting pattern across the three populations. The low-altitude population showed a significant positive Tajima’s *D* value of 2.5, *P* = 0.01. In contrast, *E. Saccharina* individuals in the high- and medium-altitude populations exhibited non-significant Tajima’s D values (high-altitude population: D = 0.04, *P* = 0.97; medium-altitude population: D = −0.6, *P* = 0.5). Furthermore, the low-altitude population showed a significant positive Fu’s Fs value (Fu’s Fs value = 9.73, P = 0.002). While Fu’s Fs statistics were similarly non-significant for high and medium altitude populations. Mismatch distribution analysis in DnaSP software produced a highly irregular, multimodal, and ragged curve (**Figure 5**) with pairwise nucleotide differences ranging from 0 to 34 among *E. saccharina* sequences.

### 3.7 Gene flow and isolation by distance

The mantel test results differed according to the level of analysis. At the individual level, the test based on 9,999 permutations revealed a statistically significant association between genetic and geographic distance (*r* = 0.27, *P* = 0.004). This individual-level pattern was also consistent with haplotype-based measures, which were relatively high (GammaST = 0.39, Nm=0.4, and N_ST_ = 0.41, Nm = 0.36). At the population level, however, the Mantel test showed no significant association between genetic and geographic distance (*r* = 0.53, *P* = 0.33).

## 4 Discussion

The present study reveals clear genetic structuring across Tanzania agroecological zones, with low-altitude populations showing greater differentiation and a stronger departure from neutrality than the high and medium altitudes (13, 32). The findings indicate that the observed genetic composition of the pest may have been influenced by ecological differences among zones, including climatic and local production conditions, as well as historical population separation over time (9, 33). Thus, the observed divergence is consistent with ecological variation across zones, as also reported elsewhere for *E. saccharina* (4, 19, 34). Similar geographic structuring has been documented in phytophagous insects where dispersal is limited and breeding habitats are localized (32, 35–37). In *E. saccharina*, the endophytic larval lifestyle, and the tendency of adults to remain close to host plants likely further reduce dispersal, thereby limiting gene flow across agroecological zones (4), reinforcing the spatial genetic structure observed in the present study.

This study hypothesized that geographic distances would influence genetic differentiation among *E. saccharina* populations in Tanzania, given the species’ limited dispersal ability, local mating behavior, and larval confinement within sugarcane stalks (38, 39). Environmental variation was also considered a potential contributor to genetic structuring (19, 40). However, the results supported a strong isolation-by-distance pattern within individuals with no strong evidence to support isolation-by-distance across the altitudes. The mantel test results indicated a scale-dependent pattern; a significant association between genetic and geographic distance was detected at the individual level, whereas no significant isolation-by-distance pattern was supported at the population level across the altitude-defined groups. These findings suggest that geographic distance alone did not fully explain population differentiation among the altitude-based populations and that ecological factors such as altitude, temperature, and relative humidity may also have contributed to the observed genetic structure (4, 41).

Previous studies reported the presence and infestation of *E. saccharina* in major sugarcane-producing companies in Tanzania (6), this study provides the first detailed genetic characterization of these populations. The closer correspondence between Tanzanian sequences and previously reported African *E. saccharina* sequences support the view that Tanzanian populations form part of the broader African mitochondrial lineage framework described elsewhere (8). At the same time, the observed sequence variation suggests that these populations are not genetically identical but instead show intraspecific divergence consistent with geographic structuring within the species (34, 42).

The medium-altitude population showed the highest number of unique haplotypes, consistent with its higher haplotype diversity relative to the other altitude-defined populations. This pattern is broadly consistent with reports that elevated haplotype and nucleotide diversity may occur in relatively stable or historically persistent populations (43, 44) and with previous evidence of mitochondrial variation in *E. saccharina* across southern and eastern Africa (8). However, because the present study was based on a small sample size and a single mitochondrial COI region, this pattern should be interpreted cautiously. In contrast, the low-altitude population contained only two haplotypes, indicating lower haplotype representation and a narrower mitochondrial diversity pattern than the other populations (13, 20, 40). Similar patterns have been reported in other insects, including recently established Asian honey bee populations in Australia (45). The low nucleotide diversity between the medium and low-altitude populations may further suggest relatively close maternal relatedness or a more recent shared maternal history between these groups (14, 34). Although the Tanzanian haplotypes were retained under study-specific labels (H1-H12) and were not directly reassigned to the nomenclature of Assefa et al. (4), the overall pattern supports earlier evidence that *E. saccharina* is not genetically homogenous across African agroecological regions.

In contrast, strong nucleotide divergence between high and medium-altitude populations indicated greater mitochondrial genetic differentiation, which may reflect reduced gene flow between these zones (43, 46, 47). The genetic isolation of high altitude may likewise be consistent with reduced effective diversity and limited connectivity (48, 49). Shared haplotypes across sampling sites suggest some degree of common ancestry in the maternal lineages (42), and phylogenetic analyses revealed clustering by altitude, a pattern frequently observed in other crop insect pests (14, 35).

The maximum-likelihood tree showed clearer mitochondrial grouping in the medium-altitude population than in the high-altitude population. Most medium-altitude sequences formed relatively well-supported clades despite their high haplotype diversity, suggesting the persistence of multiple maternal lineages in this population (34, 50). In contrast, high-altitude populations displayed weaker phylogenetic cohesion, suggesting a less distinct mitochondrial clustering pattern (16). Because the tree was used primarily to illustrate clustering among Tanzanian *E. saccharina* sequences, these results are interpreted as relative mitochondrial grouping across agroecological zones rather than evidence of a rooted evolutionary history. The prominence of medium-altitude clades may indicate that this population represents an important source of mitochondrial genetic diversity in Tanzania, although its status as an ancestral or source population remains hypothetical (35, 51).

Significant genetic differentiation across populations indicates clear population structuring and suggests reduced gene flow across altitude-defined populations (4, 49, 52). The low-altitude population was particularly distinct, consistent with patterns reported in lowland sugarcane systems in South Africa (8). Such differentiation probably reflects a combined influence of climatic variation, historical isolation, limited dispersal ability, and differences in local management practices (8, 34, 46, 51).

Although most genetic variation occurred within populations, PERMANOVA exposed statistically significant differences among populations, showing overall population structuring (33, 53). The high and medium altitude populations showed Tajima’s D values close to neutrality, with no significant departure from neutrality expectations (15, 35). Contrary to that, the low-altitude individuals showed significantly positive Tajima’s D and Fu’s Fs values, indicating a deviation from neutrality (54). However, this pattern should be interpreted cautiously because, under a small sample size and a single mitochondrial locus, positive neutrality statistics may reflect population subdivision or long-term genetic structuring and do not on their own provide definitive evidence of contraction (55). However, this pattern should be interpreted cautiously because, particularly under small sample size and a single mitochondrial locus, positive neutrality results may reflect population subdivision or long-term genetic structuring in addition to possible contraction (55). The multimodal and ragged mismatch distributions were likewise more consistent with demographic stability and/or population structuring than with a simple recent expansion model (49). Mantel test findings further indicated a scale-dependent spatial genetic pattern in *E. saccharina*, with a significant association between genetic and geographic distance at the individual level but not fully explaining the observed differentiation among populations’ scales (56, 57), and that ecological variation across altitude may also have contributed to the genetic structure of *E. saccharina* in Tanzania (58, 59). These findings point to an important role of local environmental conditions, habitat structure, and management practices in shaping the observed population structure (33, 36).

## 5 Conclusion

This study explains that *E. saccharina* populations in Tanzania are genetically diverse and structured across the three agroecological zones, with several haplotypes restricted to particular altitude-defined populations. These findings indicate altitude-associated population structuring and suggest that *E. saccharina* is not genetically homogenous across Tanzania sugarcane-growing areas. This population variation may be important for designing more targeted and locally relevant pest management strategies.

## Data Availability

The data presented in the study are deposited in the National Center for Biotechnology Information (NCBI) repository under accession numbers PZ306143–PZ306146 and PZ306908–PZ306927.
